# Statin Treatment and Mortality in Bacterial Infections – A Systematic Review and Meta-Analysis

**DOI:** 10.1371/journal.pone.0010702

**Published:** 2010-05-19

**Authors:** Linda Björkhem-Bergman, Peter Bergman, Jan Andersson, Jonatan D. Lindh

**Affiliations:** 1 Division of Clinical Pharmacology, Department of Laboratory Medicine, Karolinska University Hospital Huddinge, Karolinska Institutet, Stockholm, Sweden; 2 Division of Clinical Microbiology, Department of Laboratory Medicine, Karolinska University Hospital Huddinge, Karolinska Institutet, Stockholm, Sweden; 3 Division of Infectious Diseases, Department of Medicine, Karolinska University Hospital Huddinge, Karolinska Institutet, Stockholm, Sweden; Columbia University, United States of America

## Abstract

**Background:**

Several studies have reported improved survival in severe bacterial infections among statin treated patients. In addition, statins have been ascribed beneficial anti-inflammatory effects. The aim of this study was to evaluate the effect of statin-treatment on mortality in patients with bacterial infections, by means of a systematic review and a meta-analysis.

**Methodology and Principal Findings:**

Studies investigating the association between statin use and mortality in patients with bacterial disease were identified in a systematic literature review and a meta-analysis was performed to calculate the overall odds ratio of mortality in statin users. The literature search identified 947 citations from which 40 relevant studies were extracted. In all, 15 studies comprising 113 910 patients were included in the final analysis. Statin use was associated with a significantly (p<0.0001) reduced mortality in patients suffering from bacterial infections (OR 0.52, 95% CI 0.42–0.66). However, all studies included were of observational design and funnel plot analyses indicated influence by a possible publication bias (Egger's bias test p<0.05). When a precision estimate test was used to adjust for publication bias the effect of statin treatment was no longer significant, with an OR of 0.79 (95% CI 0.58–1.07).

**Conclusion/Significance:**

According to the meta-analysis of observational studies presented here, patients on statin therapy seem to have a better outcome in bacterial infections. However, the association did not reach statistical significance after adjustment for apparent publication bias. Thus, there is a great need for randomised controlled trials investigating the possible beneficial effect of statins in bacterial infections.

## Introduction

Statins (HMG-CoA reductase inhibitors) are today some of the most prescribed drugs in the world due to their beneficial effects on cardiovascular disease. During recent years statins have been ascribed additional beneficial effects on the outcome of other diseases, such as cancer and infections [1], [2]. Some of these “pleiotropic effects” might be explained by a potential anti-inflammatory effect of statins [3], [4]. In the PRINCE-study pravastatin treatment reduced the levels of C-reactive protein (CRP) in a cholesterol independent way, although the clinical significance of this reduction remains unresolved [5]. Beneficial effects of statin treatment in transplanted patients have been reported and suggest an immunomodulatory effect [6], [7] although a recent Cochrane review failed to show a significant reduction in all-cause mortality among statin treated renal transplant patients [8].

A number of epidemiologic studies support that statin treatment is associated with a better prognosis in severe bacterial infections [1]. However, there is a lack of randomised controlled trials that prospectively investigate the effect of statin treatment in patients diagnosed with severe infections.

In a recent systematic review by Kopterides and Falagas [1] 22 original studies on the role of statins in infections were identified. These studies yielded heterogeneous results and were not always powered to provide precise quantitative estimates of the potential statin effect. To provide a summarized estimate of the proposed statin effect Tleyjeh et al performed a meta-analysis demonstrating that statin use was associated with a beneficial effect in treating and preventing infections [9]. However, meta-analyses of observational studies are sensitive to influences by confounding and other sources of bias and the result of such analyses should be interpreted with great caution. Here, we present the results of a new systematic review and meta-analysis, expanding on previous findings by inclusion of novel studies and older studies that were not included in the meta-analysis by Tleyjeh et al. In addition, we have performed an in-depth analysis of potential bias sources, providing quantitative estimates of their influence on the meta-analysis results. Our primary objective was to summarize and critically evaluate the current knowledge on statin treatment as a *therapeutic agent* during bacterial infections. Studies investigating the prophylactic effects of statins were excluded in our analysis. However, to give a better over-view of all studies published in this field we chose to present data from *all* relevant studies found in our literature search, i.e also the prophylactic studies excluded in the meta-analysis.

## Methods

### Search strategy

The literature search was conducted in PubMed and Embase (finished April 2010). Searches were not restricted to time or language.

In PubMed the following terms for statins as MeSH or text word (Hydroxymethylglutaryl-CoA Reductase Inhibitors OR Hydroxymethylglutaryl-CoA Reductase Inhibitors [Pharmacological Action] OR HMGCoA reductase inhibitors OR simvastatin OR lovastatin OR pravastatin OR fluvastatin OR atorvastatin OR cerivastatin OR rosuvastatin OR pitavastatin OR statins) were combined with (bacteremia OR sepsis OR pneumonia OR infections), as both MeSH or text word.

In Embase the following terms for statins (hydroxymethylglutaryl coenzyme a reductase inhibitor OR simvastatin OR lovastatin OR pravastatin OR fluvastatin OR atorvastatin OR cerivastatin OR rosuvastatin OR pitavastatin OR statins) were combined with terms for bacterial infections (bacteremia OR sepsis OR pneumonia OR bacterial infection) and terms for the study types (prevention study OR pilot study OR observational study OR experimental study OR controlled study OR comparative study) all as Emtree preferred terminology.

In cases of missing data authors were contacted for additional information or unpublished data.

### Study selection criteria

The aim of the present investigation was not to study the potential preventive effect of statins against infection but rather to evaluate the influence of statin treatment on the disease progress and prognosis in bacterial infection. In accordance with this included studies had to meet the following criteria: (1) retrospective or prospective studies investigating the outcome in bacterial infections in statin treated versus non-statin treated patients (2) all included patients had to have a bacterial infection such as pneumonia, sepsis, bacteraemia or symptoms of a severe bacterial infection. (3) All-cause mortality data or infectious mortality data in statin treated versus non-statin treated subjects had to be presented. If effect estimates adjusted for potential confounders were not presented the authors were contacted with a request for adjusted data. Statin use was defined as current statin use at time of hospital admission. Studies addressing viral infections such as HIV or CMV were excluded.

### Data extraction

One reviewer (LBB) initially evaluated all the abstracts from the literature search and extracted potentially relevant studies addressing the association between statin exposure and the outcome of bacterial infections. These studies were retrieved and evaluated by two independent reviewers (LBB and PB). Studies fulfilling the inclusion criteria of the meta-analysis were extracted and summarized independently by the two reviewers (LBB and PB). Recorded data included characteristics of the studies, demographic data, and outcome in statin users and non-users with severe infections. Any discrepancies were resolved by consensus.

### Statistical methods

The primary effect measure used in the meta-analysis was the infectious mortality or odds ratio (OR) for in-hospital or all-cause 30 day mortality. If both all-cause and infectious mortality was available, the latter was used as primary effect measure. When available, ORs adjusted for potential confounders (age, sex, disease severity, comorbidities, comedication etc.) were used. When adjusted OR was not presented the authors were contacted for additional information. If no adjusted OR-values could be obtained the study was excluded from the main analysis.

Odds ratios from all included studies were pooled in a meta-analysis weighting the individual studies according to their log-transformed inverse variance. Homogeneity among studies was tested by means of Cochran's Q test and calculation of the variation across studies attributable to heterogeneity rather than chance (I^2^). When substantial heterogeneity was demonstrated (defined as a Cochran Q test p value <0.1 or a I^2^ value >25%) a random-effects model was used to calculate the overall effect, otherwise a fixed-effect model was used. In a secondary analysis, we repeated the meta-analysis after addition of the studies where only unadjusted odds ratios were available, excluded in the main analysis.

Sensitivity analyses were performed to assess the influence of various study characteristics on the observed statin effect. Both studies with adjusted and studies with unadjusted odds ratios were included in the sensitivity analyses. Firstly, the possible influence of publication bias was graphically evaluated by means of a funnel plot where log-transformed odds ratios were plotted against standard errors. The presence of funnel plot asymmetries was formally analysed using Egger's bias test. In addition, the precision-effect test (PET) was used to calculate an overall OR adjusted for observed funnel plot asymmetries indicative of publication bias [10]. In this test, the normalized effect estimate (OR divided by its standard error) is regressed on the inverse standard error of the effect, whereby the slope of the regression lines offers a logarithmised estimate of the pooled OR adjusted for any observed association between effect size and study precision.

Secondly, the influence of a range of study-level and aggregated individual-level parameters on the observed statin effect was investigated by means of meta-regressions. In these analyses, the odds ratio from each study was regressed on the potential confounders in univariable and multivariable weighted linear regressions weighted according to the inverse standard error and the residual between-trial variance. Independent variables included in the multivariable analysis were chosen by means of backward selection with successive exclusion of the least significant variable until only significant predictors of study outcome remained in the model. In total, twelve dichotomous and four continuous parameters were investigated in these analyses. The dicothomous parameters (coded as 1 for yes and 0 for no) were prospective study design, case-control design (as opposed to cohort design) Asian, European, or North-American setting (Australian studies were coded 0 for each of these; no African studies were identified), industry-sponsored or industry-independent studies (studies with undisclosed financing were coded 0 for both), infection-related mortality as outcome (as opposed to all-cause mortality), in-hospital mortality (as opposed to e.g. 30-day mortality), infection-related mortality as outcome (as opposed to all-cause mortality), statistical adjustment for potential confounders performed, and pneumonia or sepsis/bacteremia as requirement for inclusion. Continuous variables were the impact factor of the journal where the study was published, standard error of the association measure (OR), patient age (per year), and the proportion of included patients being male (ranging from 0 in a study with only female subjects to 1 in an exclusively-male study).

Thirdly, the potential influence of unknown confounders (residual confounding) was investigated by means of a rule-out approach described by Schneeweiss [11]. This approach stipulates the influence of a hypothetical confounder and determines what characteristics this confounder must have in order to fully account for the observed association between statin use and mortality. The hypothetical confounder is characterised by its association to statin use (OR_EC_, odds ratio of exposure to the confounder in statin non-users vs statin users) and its association to the outcome (RR_CO_, relative risk of outcome in individuals exposed to the confounder vs non-exposed). For this analysis, the absolute risk in the pooled control group was used for conversion of odds ratio to relative risk. Separate analyses were performed to demonstrate what levels of OR_EC_ and RR_CO_ that would be required to fully explain the observed association between statins and mortality before and after adjustment for publication bias as described above.

Finally, the overall effect of adjusting for potential individual-level confounders was investigated by calculating the mean ratio of adjusted odds ratio over unadjusted odds ratio in the subset of studies where both effect measures were available.

In all analyses except the Cochran's Q test, results associated with p-values <0.05 (two-sided test) were considered statistically significant. The statistical analyses were performed using StatsDirect statistical software version 2.7.2 (StatsDirect, Sale, Cheshire, UK).

## Results

### Identification of studies

**Figure 1:**
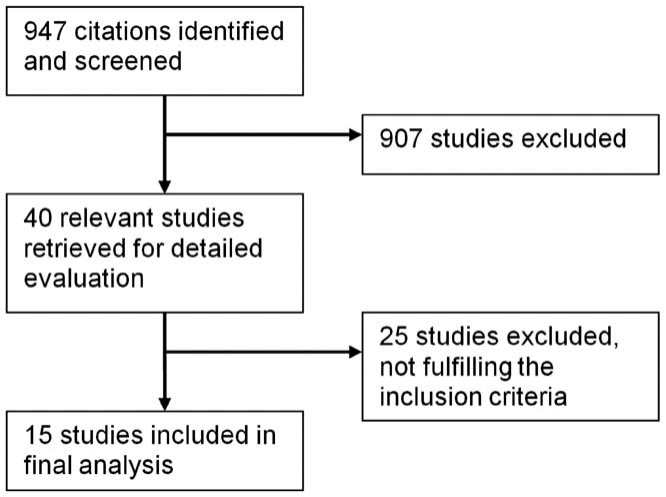
Flow diagram showing the number of citations identified, retrieved and included in final analysis.

The literature search resulted in 947 citations, 39 of which addressed statin-treatment and the outcome of bacterial infections (Figure 1). This was in accordance with the literature identified in earlier systematic reviews [1], [9] but with addition of several novel studies. One article comprised both a case-control study and a cohort study [12] and was therefore divided into two studies. We defined the studies as either prophylactic (studies of association between statin use and *risk of acquiring infection*) or therapeutic (studies of association between statin use and *outcome of manifest infection*).

Evaluation of the 40 studies showed that 21 studies [12], [13], [14], [15], [17], [18], [19], [20], [21], [22], [23], [24], [25], [26], [27], [28], [29], [30], [31], [32], [33] were investigating the prophylactic effect of statin treatment on infections and 19 investigating the therapeutic effect [12], [16], [34], [35], [36], [37], [38], [39], [40], [41], [42], [43], [44], [45], [46], [47], [48], [49], [50]. All the prophylactic studies were excluded from the meta-analysis since the patients did not have a bacterial infection at baseline in these studies. One study investigated the therapeutic effect of statins in patients with multiple organ dysfunction failure (MODS) but since the MODS was due not only to sepsis or pneumonia but also to cardiac causes or stroke this study was excluded [47]. One study investigated the therapeutic effect of statins in patients with fever >38°C at admission to hospital but since there was documentation of bacterial infections in only 16% of the patients this study was also excluded [36].

One study did not distinguish between influenza and/or bacterial pneumonia and since the cause of death in influenza is often due to secondary bacterial infections this study was included [43]. In the study by Donnino and co-workers [37] all patients had a diagnosis consistent with infections (pneumonia, sepsis, pyelonephritis) or symptoms of severe infections (altered mental status, shortness of breath etc). Since the majority of the patients were assumed to suffer from a bacterial infection, and that this was the cause of death, this study was included.

Two of the therapeutic studies were excluded since only unadjusted OR values for mortality in bacterial infections were available [34], [42]. In the study by Frost et al the statin-exposed group (n = 3578) was subdivided into statin user with a low dose (n = 238) and a moderate dose (n = 3340). Only the data from the statin users with a moderate dose (≥4 mg/day) was included [12].

### Results from the included studies (Table S1)

Fifteen studies comprising 113 910 patients (8611 statin-users and 105 299 non statin-users) fulfilled the inclusion criteria for the meta-analysis and were included in the final analysis (Table S1).

Table S1 presents the characteristics of the studies included in the meta-analysis. All studies were published between 2001 and 2009 and the mean age in the studies varied between 48–75 years. In 5 of the 15 included studies the type of statins used was described. Several different types of statins were used in all these studies, although simvastatin was usually the most common one. In two studies estimates of infection-related mortality could be obtained [12], [50]. The factors adjusted for in the different studies included age [12], [16], [35], [38], [41], [43], [45], [46], [48], [49], sex [12], [16], [37], [38], [41], [43], [45], [46], [48], [49], co-morbidity [35], [37], [38], [41], [44], [46], [48], [49], severity of illness [16], [35], [37], [38], [44], [46], [48], [49], co-medication [35], [41], [43], [45], [46], [48], [49], marital status [43], [45], [49], smoking [35], [41], [46], alcohol-related conditions [44], [48], [49], characteristic of infections [38], [48], quality of care [37], [38], [41], [44], [48], [49] and ethnicity [43], [45].

Statin-users in all studies were on current statin treatment at the time of hospital admission and in five studies all patients defined as statin users had to be on statin treatment during the whole in-hospital stay [37], [39], [40], [41], [50]. In 13 studies the time exposure for statin treatment before hospital admission was unknown. In the case-control study of Frost et al statin-users had been taking statins for at least 3 months [12] and in the study by Yang et al statin exposure was at least 1 month in all statin users [50].

In the articles addressing “bacteraemia” this entity was defined as the presence of at least one positive blood culture of a clinically relevant pathogen. In three of the four bacteraemia studies, patients should also have symptoms of infection. In the articles addressing “sepsis” this diagnosis was defined according to ICD-9 criteria for sepsis and/or the sepsis criteria proposed by American Collage of Chest Physicians and the Society of Critical Care Medicin (ACCP-SCCM) [51]. The patients in the sepsis studies generally had a more severe disease compared to the patients in the bacteraemia studies, and the mean mortality in the sepsis studies was 34% compared to 21% in the bacteraemia studies. The mean mortality in the pneumonia studies was 11% and in the studies addressing any infection it was 6%.

### Results from prophylactic studies (Table S2)

Many of the originally selected studies were investigating the *prophylactic* effects of statins on infection and thus did not meet the inclusion criteria of our meta-analysis. However some were of high-quality and with potentially valuable results and therefore we chose to present data from all excluded studies in a separate table (Table S2). The article by Frost et al comprised two studies, where the case-control study were included in the meta-analysis (presented in Table S1) and the cohort study was excluded and therefore presented in Table S2 [12].

We identified 9 studies investigated the association between statin use and risk of developing pneumonia or respiratory infections [12], [17], [19], [23], [24], [27], [29], [30], [33], 5 showing some beneficial effect and 4 revealing no significant effect.

In the large cohort study by Hackam et al and in the prospective cohort study by Gupta et al a significantly decreased risk of sepsis and fatal sepsis in statin users could be observed [20], [21].

In the two small RCTs by Tseng et al and Montaner et al there was no convincing association of statin use and a beneficial outcome on infection post subarachnoidal haemorrhage or stroke but the limited number of patients included makes the results less reliable [26], [32].

Five studies investigated the association between statin treatment and post operative infections after cardiac surgery [14], [25], [31], herniorraphy [22] or any elective surgery [15]. Only one of these studies showed a marginally favourable effect of statin and the over-all impression from these studies is that statins have no convincing prophylactic effect on post-operative infections.

Of all the 40 studies described here (table S1 and S2), 22 studies showed a significantly favourable association between statin therapy and the outcome of bacterial infection, 16 showed no significant association and 2 showed unfavourable association with statin exposure.

### Meta-analysis of therapeutic studies

**Figure 2:**
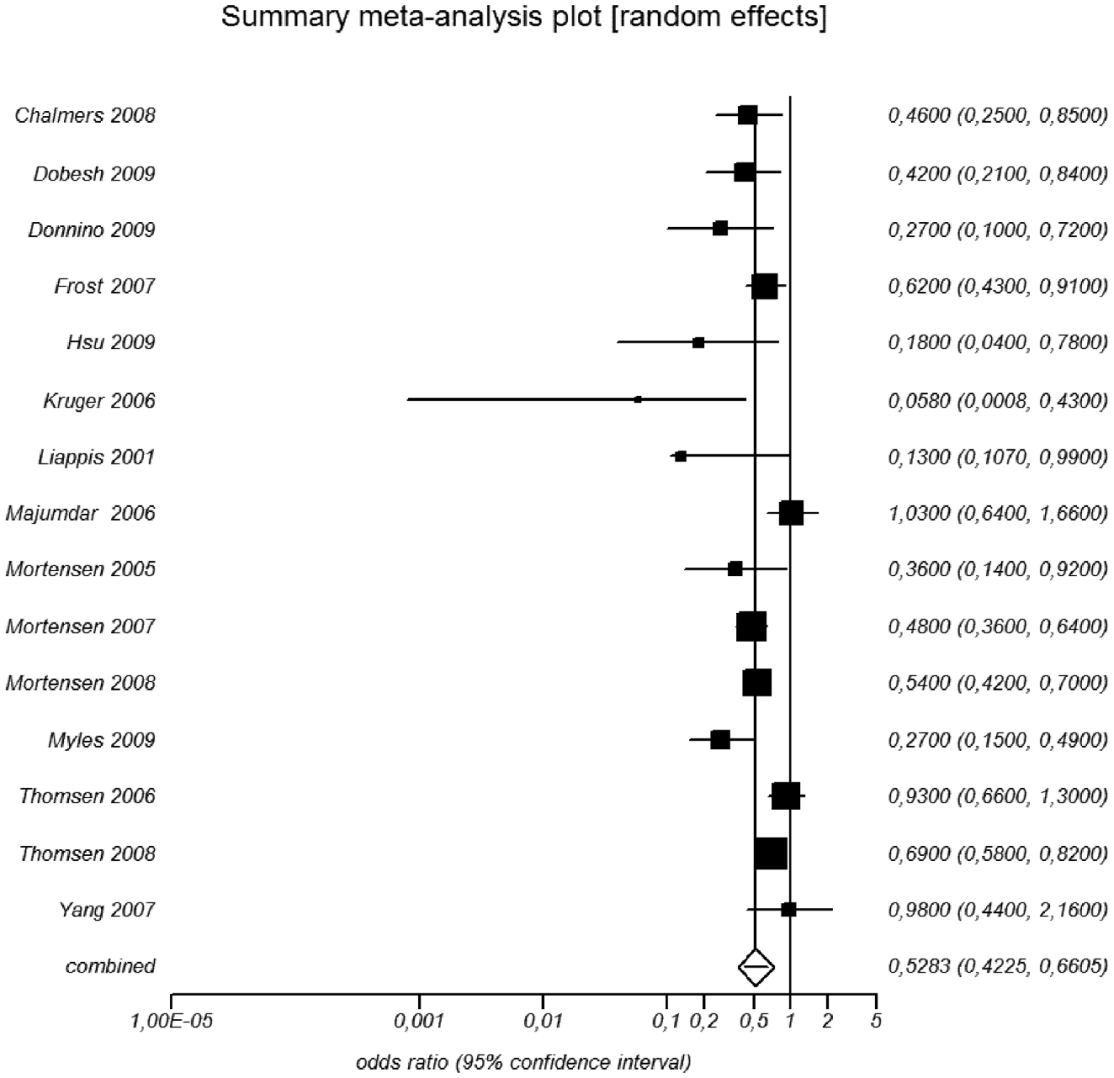
Forest plot of all included studies in the meta-analysis. Estimated OR in statin-users vs non-users for infectious/30-day/in-hospital mortality among patients diagnosed with a severe bacterial infection. Brackets denote 95% confidence intervals.

There was evidence of a substantial between-studies heterogeneity in the overall meta-analysis (Cochran Q test p = 0.001, I^2^ = 60%) and consequently a random-effects model was used in the meta-analysis. A forest plot of the analysis of all 15 included studies is presented in figure 2. Statin use was associated with a significantly (p<0.0001) reduced mortality in patients suffering from bacterial infections (OR 0.52, 95% confidence interval (CI) 0.42–0.66). Inclusion of two additional studies where only unadjusted values were available [34], [42] had no influence on the outcome of the meta-analysis (OR 0.53, 95% CI 0.43–0.65).

### Sensitivity analyses

**Figure 3:**
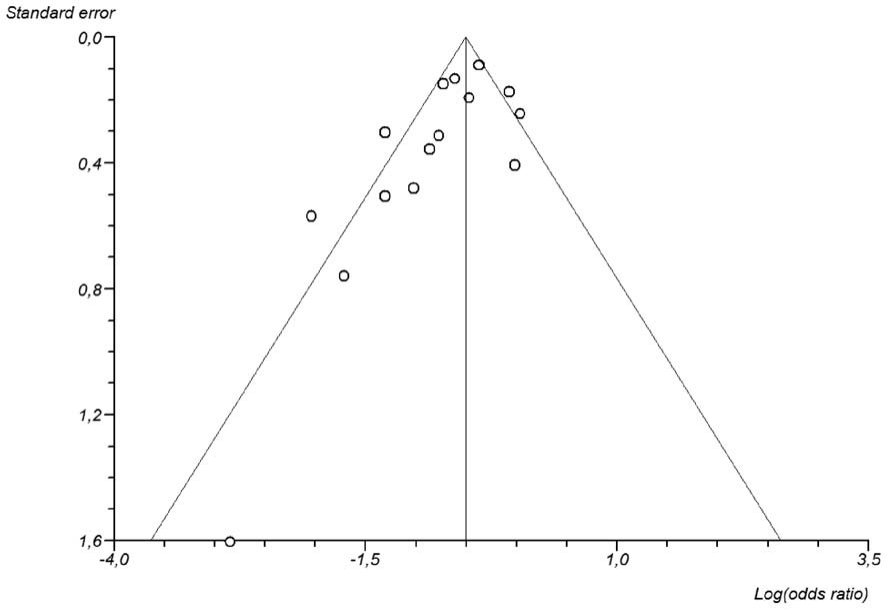
Funnel plot of the association between the estimated effect size and its standard error in individual studies. The stronger association between statin use and mortality seen in studies with lower precision (large standard error) is suggestive of publication bias with selective publication of studies with favourable results.

A funnel plot of the studies included in the overall analysis is presented in figure 3. From this plot it is evident that studies with a lower precision (larger standard error) were more likely to present large statin effects, a pattern indicative of publication bias. This was confirmed by a significant Egger's bias test (p<0.05). When the precision estimate test was used to adjust for publication bias, the overall OR was altered to 0.79 (95% CI 0.58–1.07).

The outcome of the univariable and multivariable meta-regressions are presented in table S3. In the univariable analyses, the only factors significantly associated with study outcome was the prospective study design and industry sponsorship. Since the number of potential confounders addressed was too large for the initial multivariable model, age, impact factor, case-control design, and European/North-American origin were excluded from the multivariable analysis. These factors exhibited high levels of correlation with other included variables and excluding them effectively reduced the co-linearity of the model. After exclusion of non-significant predictors, the final model included seven potential confounders: prospective study design, adjustment for potential confounders, industry-independent funding, Asian setting, in-hospital mortality and infection-related mortality as outcome, and standard error of the effect estimate. A larger standard error, adjustment for confounding and the use of infection-related mortality as study outcome were associated with lower odds ratios while the remaining four factors were associated with higher odds ratios.

**Figure 4:**
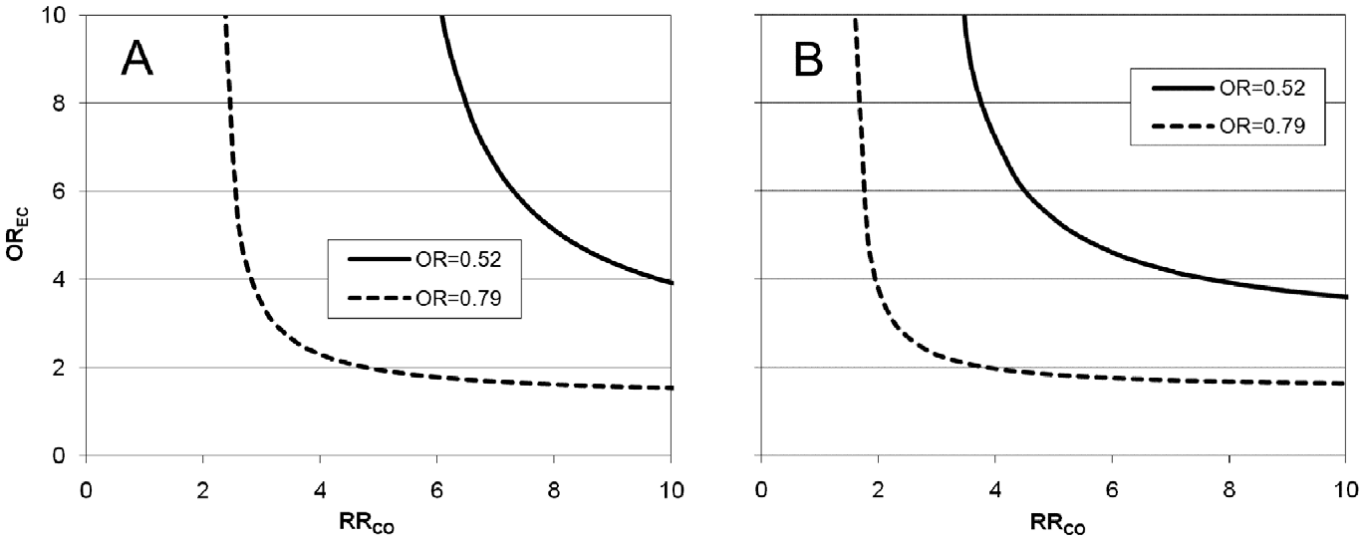
Modelled influence of a hypothetical dichotomous confounder present in 20% (panel A) and 50% (panel B) of the study population, unaccounted for in the adjustments already performed in the individual studies. The graphs indicate what combinations of OR_EC_ and RR_CO_ that would be necessary for the confounder to fully account for the observed association between statin use and mortality before (OR = 0.52) and after (OR = 0.79) adjustment for publication bias. Abbreviations: OR_EC_, odds ratio of exposure to the confounder in statin non-users vs statin users; RR_CO_, relative risk of mortality in individuals exposed to the confounder vs non-exposed.

The results of the residual confounding analysis are presented in figure 4. Panel A refers to a confounder with a prevalence of 0.20 and at this prevalence level even a very strong confounder causing a ten-fold increased mortality risk would have to be severely imbalanced between statin users and non users (OR_EC_ = 3.9) in order to fully account for the observed unadjusted OR of 0.52. Adjusting for apparent publication bias (adjusted OR = 0.79) reduced the demands on the hypothetical confounder. Nevertheless, a confounder that increase the mortality risk a full four times would still have to be distinctly imbalanced (OR_EC_ = 2.3) in order to explain the adjusted statin effect. For a very common confounder with a prevalence of 0.50, weaker associations with statin use and/or mortality would be sufficient to explain the observed association between statin use and mortality (figure 4, panel B). However, at this prevalence level the confounder would still have to be both quite imbalanced and increase the mortality risk several fold in order to account for the observed OR, even after taking publication bias into account.

When comparing adjusted vs unadjusted ORs within studies, the former were on average 6% lower, indicating a modest influence of the individual-level confounders addressed in these studies.

## Discussion

The findings in this systemic review and meta-analysis show a possible association between statin treatment and reduced mortality rate after having been diagnosed with bacterial infections. In addition, the combined results from the prophylactic (excluded) studies presented in Table S2 imply that a possible beneficial *prophylactic* effect of statins in prevention of sepsis and pneumonia cannot be ruled out. In contrast, there appears to be weak evidence for statins in the prevention of post-operative infections (Table S2).

According to the meta-analysis presented here, statin use is associated with an almost 50% lower odds of death during bacterial infections. Although this estimate is mainly based on data adjusted for potential confounders such as differences in age, sex, disease severity and comorbidities, the sensitivity analysis clearly indicates that the result may still be influenced by other sources of bias. One such factor is likely to be publication bias due to selective publishing of results in favour of a pronounced statin effect. Unlike Tleyjeh and co-workers [9], we were able to demonstrate a significant influence by publication bias (Egger's bias test p<0.05). Notably, the previous meta-analysis included both prophylactic and therapeutic studies.

Based on our finding, we further attempted to adjust our results for the observed signs of publication bias, discovering that approximately half the observed statin effect could be attributable to publication bias.

To identify other factors of potential importance for the observed statin effect, we investigated the association between a range of study characteristics and the outcome of individual studies. Factors that were predictive of a smaller statin effect in a multivariable analysis were prospective study design, industry-independent funding, Asian study setting and a short follow-up time (in-hospital mortality). The mechanisms underlying these associations are not clear, but the weaker association seen in studies with a prospective study design may indicate additional bias sources in studies with retrospective design, leading to inflated association strength estimates in these studies. Similarily, industry sponsorship is a factor known to influence the outcome of clinical trials, with a bias in favour of products marketed by the supporting organisation [52]. The lower odds ratio associated with in-hospital mortality rather than 30-day mortality could reflect a protective effect of statins most pronounced after the acute phase of infection, but the limited information does not allow any definitive conclusions regarding the time-course of the proposed statin effect. Theoretically, the reduced OR (closer to null) in the Asian studies could represent an influence of pharmacogenetic or environmental factors, but until more data from Asian patient populations are available, this association should be interpreted with great caution. Factors predictive of a stronger association between statin use and mortality in patients with infection were the use of infection-related mortality rather than all-cause mortality as clinical endpoint and the application of statistical adjustment for potential patient-level confounders. The former factor could speak in favour of a causal relationship between statin exposure and the clinical course of manifest infection, since such a protective effect may be most evident on infection-related mortality, undiluted by other causes of death. The stronger association in studies adjusting for potential confounders may seem surprising if adjustment is primarily seen as a means of removing artificial associations based on confounding. However, confounding factors can also work in the opposite direction by attenuating true associations. In this situation, the adjustment is expected to strengthen the association as seen in the sensitivity analysis. The association between adjustment and a lower OR is further supported by the within-studies analysis, where adjusted ORs on average were 6% lower than their unadjusted counterparts.

All the included studies were of observational design, and the patients were non-randomly assigned to statin therapy. Since some factors underlying the decision to treat a patient with statins are likely to be unknown or unavailable for analysis, it is not possible to fully adjust the results for confounding. For example, the results from this meta-analysis could be influenced by the “healthy-users” effect, i.e. a situation where statin-users systematically care more for their health, have a higher socioeconomic status and other characteristics making them more likely to survive a severe infection. This is highlighted in an interesting article of Dormuth et al where statin adherence is reported to be associated with a significant decreased risk of developing diseases unrelated to the known biological effect of statins as well as a decreased rate of having accidents [53]. In addition, statin adherence was associated with an increased likelihood of using medical screening services [53]. On the other hand, patients taking statins most probably have a history of cardiovascular disease or risk factors for cardiovascular disease and could therefore be expected to have a poorer prognosis associated with severe infections putting a strain on the cardiovascular system.

To investigate the potential influence of uncontrolled confounding on the results, we calculated the magnitude of association with statin use and mortality that a hypothetical confounder would need to fully account for the apparent statin effect. This analysis showed that only a very strong confounder would be able to explain the statin association seen in the main analysis. After adjustment for publication bias, a more modest confounder could be sufficient. However, a highly prevalent confounding factor that doubles the mortality risk would still have to be quite imbalanced between the statin non-users and the statin users (OR of exposure to confounder approaching 4) while a more balanced confounder (OR = 2) would have to increase mortality by 4-fold in order to account for the observed association between statins and mortality. Hence, it seems unlikely that the observed association is entirely due to confounding and that the statins are completely devoid of a causal influence on mortality in severe infection. In addition, it should be noticed that the confounding model employed stipulates a cofounder entirely independent of the factors adjusted for in the individual studies. If, as may often be the case, the unknown confounder is correlated to the confounders already adjusted for, its impact could be much smaller than the model indicates.

The reduced mortality in severe bacterial infections might be explained by the reported anti-inflammatory and immunomodulatory effects of statins [3], [4]. By inhibiting the HMGCoA-reductase, statins inhibit the synthesis of isoprenoid units, necessary for the activity of a number of proteins, such as the GTP-binding protein Rho. Statins may thus affect transcription factors such as NFκB and AP-1, leading to a decreased synthesis of pro-inflammatory cytokines such as IL-1 and IL-6 [4]. Moreover, statins have also been reported to induce caspase-dependent apoptosis in smooth muscle cells [4], which leads to less inflammation by avoiding the necrotic cell-death pathway. Despite the multitude of possible immunomodulatory actions exerted by statins the protective immune response against pathogens appear to remain intact. Thus, it could be speculated that statins strengthen immunity to pathogens while simultaneously dampen the inflammatory response connected to bacterial infections and sepsis. Interestingly, *in vitro* experiments have suggested that statins specifically inhibit proinflammatory Th1- and Th17-cells shifting the T-cell population towards an anti-inflammatory profile dominated by IL-10 producing T-regulatory cells [54], [55]. However, the *in vivo* mechanism behind the reported anti-inflammatory effects of statins is not fully elucidated. Notably, the concentrations used in most *in vitro* experiments are up to 1000 times higher than what is detected in human plasma during statin treatment [54], [55], [56].

It has been reported that both the lipid-lowering and anti-inflammatory effect of statins differ between individual substances in the drug class [57], [58]. It would have been of great interest to make a subgroup analysis comparing different statins with regard to their effect on mortality. However, this was not possible since all studies were of observational design, lacking allocation to a specific type of statin. Moreover, information on the individual statins used was only provided in 5 of the 15 studies.

Even though the results indicate that statins may improve the prognosis in bacterial infection, the magnitude of this effect is not easily estimated due to potential bias. However, we can conclude that statin treatment does not appear to be associated with any unfavourable outcome on mortality in severe bacterial infections. To fully compensate for different bias-effects inherent to observational studies, large randomised controlled trials (RCT) testing the hypothesis that statin treatment has beneficial effects on the outcome of infections are highly warranted. In fact, several such studies are ongoing according to www.clinicaltrials.gov and an additional RCT will commence at our center during 2010 at Karolinska University Hospital Huddinge, Sweden (personal communication: Dr Christer Lidman and Prof Jan Andersson).

In conclusion, we believe that our meta-analysis provide a solid rationale for future interventional randomized controlled trials investigating the hypothesis that statins improve the clinical outcome of infectious diseases.

## Supporting Information

Table S1 Characteristics of included studies.
(0.06 MB DOC)

Table S2 Characteristics of excluded studies.
(0.09 MB DOC)

Table S3 Influence of study characteristics on estimated association between statins and mortality.
(0.06 MB DOC)
