# Supplementary material for: Statin Treatment and Mortality in Bacterial Infections – A Systematic Review and Meta-Analysis
Source: PLoS One. 2010 May 19;5(5):e10702. doi: 10.1371/journal.pone.0010702 (PMC2873291; doi:10.1371/journal.pone.0010702)
Supplement: Table S1 — Characteristics of included studies. (0.06 MB DOC) [file pone.0010702.s001.doc]

Table 1. Characteristics of included studies

| **Study, year** | **Infections** | **Study design** | **Country** | **Male** | **Mean**  **age** | **Statin users (n)** | **Non-statin**  **users (n)** | **Adj OR** ^2^  **(95% CI)** |
| --- | --- | --- | --- | --- | --- | --- | --- | --- |
| Chalmers (2008) [35] | pneumonia | observational prospective cohort | UK | 50% | 66 ^1^ | 257 | 750 | **0.46** (0.25-0.85) |
| Dobesh (2009) [16] | sepsis | observational retrospective cohort,  (patients admitted to ICU) | USA | 53% | 66,5 | 60 | 128 | **0.42** (0.21-0.84) |
| Donnino (2009) [37] | any infections | observational retrospective cohort | USA | 48% | 61 | 474 | 1562 | **0.27**  (0.10-0.72) |
| Frost (2007) case-control [12] | pneumonia / influenza | case-control study | USA | 47% | 48-58 ^1^ | 3340 | 50955 | **0.62** (0.43-0.91) ^3^ |
| Hsu (2009) [38] | bacteraemia | observational retrospective cohort  (transplanted patients) | USA | 60% | 51 | 78 | 230 | - 1. 0.04-0.78) |
| Kruger (2006) [39] | bacteraemia | observational retrospective cohort | Australia | ND | ND | 56 | 372 | **0.058** (0.0008-0.43) |
| Liappis (2001) [40] | bacteraemia | observational retrospective cohort | USA | 100% | 63 | 35 | 353 | **0.13** (0.11-0.99) |
| Majumdar (2006) [41] | pneumonia | observational prospective cohort study | Canada | 53% | 75 ^1^ | 325 | 3090 | **1.03** (0.64-1.66) |
| Mortensen (2007) [45] | sepsis | observational retrospective cohort | USA | 99% | 74 | 480 | 2538 | **0.48** (0.36-0.64) |
| Mortensen (2008) [43] | pneumonia | observational retrospective cohort | USA | 99% | 75 | 1567 | 7085 | **0.54** (0.42-0.70) |
| Mortensen (2005) [44] | pneumonia | observational retrospective cohort | USA | 79% | 60 | 110 | 677 | **0.36** (0.14-0.92) |
| Myles (2009) [46] | pneumonia | observational retrospective cohort | UK | ND | ND | 177 | 3504 | **0.27** (0.15-0.49) |
| Thomsen (2008) [49] | pneumonia | observational retrospective cohort | Denmark | 53% | 73 ^1^ | 1372 | 28528 | **0.69** (0.58-0.82) |
| Thomsen (2006) [48] | bacteraemia | observational prospective cohort study | Denmark | 55% | 65-79 ^1^ | 176 | 5177 | **0.93** (0.66-1.32) |
| Yang (2007) [50] | sepsis | observational retrospective cohort | Taiwan | 55% | 64 | 104 | 350 | **0.98**  (0.44-2.16) |

Abbreviations: ND - no data, ICU – intensive care unit, Adj – adjusted, CI – confidence interval

^1^ median age or median age interval

^2^ OR of infectious mortality [12,50], in-hospital mortality [16,37,39,40,41], 30-day all cause mortality [35,43,44,45,46,48,49,50], or 15 day all-cause mortality [38].

^3^ Only the data from statin users with a moderate dose of statins (≥ 4 mg / day) was included. Daily dose was defined as mean mg / day for a 3 months – 1 year period following initiation of statin therapy [12]
