# Supplementary material for: Statin Treatment and Mortality in Bacterial Infections – A Systematic Review and Meta-Analysis
Source: PLoS One. 2010 May 19;5(5):e10702. doi: 10.1371/journal.pone.0010702 (PMC2873291; doi:10.1371/journal.pone.0010702)
Supplement: Table S2 — Characteristics of excluded studies. (0.09 MB DOC) [file pone.0010702.s002.doc]

Table 2: Characteristics of excluded studies

| **Study, (year)** | **Infection** | **Country** | **Male** | **Mean Age** | **Study design** | **SU**  **(n)** | **NSU**  **(n)** | **Outcome on infections** |
| --- | --- | --- | --- | --- | --- | --- | --- | --- |
| Almog (2004) [34] | pneumonia, sepsis, UTI, cellulitis | Israel | 49% | 70 | Prospective, observational population-based | 82 | 279 | Relative risk in septic patients of developing severe sepsis: 0.13 (95% CI, 0.03- 0.52),  In-hospital death in septic patients: unadj OR 0.40 (95% CI 0.12-1.38), our calculation based on extracted crude data. |
| Almog [13]  (2007) | all infections | Israel | 63% | 65 | Prospective, observational population-based | 5698 | 5664 | Infection-related mortality:  relative risk 0.22 (95% CI 0.17-0.28) |
| Coleman [14]  (2007) | all infections, postop. | USA | 71% | 66 | retrospective cohort (post op: cardiac surgery) | 1248 | 686 | post op infection, adjusted OR: 0.67 (95% CI 0.46-0.99, p=0.04) |
| Daneman (2009) [15] | Wound infections, postop. | Canada | 55% | 73 | retrospective matched cohort (post op: wound infections) | 53 565 | 53 565 | Surgical site infections post op adj OR: 1.00 (95% CI 0.95-1.04) |
| Dublin [17]  (2009) | pneumonia | USA | 51% | 77* | Population-based case-control | 508 | 2852 | Pneumonia, adjusted OR: statin for secondary prevention 1.25 (95% CI 0.94-1.67) with no such indication 0.81 (95% CI 0.46-1.42) |
| Fernandez [18] (2006) | ICU-aquired infections | Spain | 70% | 62 | retrospective cohort (ICU patients) | 38 | 400 | ICU-aquired infection rate: non-significantly lower (29% vs 38%). Hospital mortality, adjusted OR: 2.3 (95% CI 1.08-4.89) |
| Fleming (2009) [19] | respiratory infections,  urinary tract infections | UK | 45% | 55-64* | population-based retrospective cohort | 267 622  (person years) | 61 259  (person years) | Influenza-like illness adj OR: 1.05 (95% CI 0.92-1.20),  acute bronchitis adj OR: 1.08 (95% CI 1.01-1.5)  Pneumonia adj OR: 0.91 (95% CI 0.73-1.13)  Acute resp infection adj OR: 1.03 (95% CI 0.98-1.07)  Urinary tract infections adj OR: 0.91 (95% CI 0.85-0.98) |
| Frost, the cohort study [12] (2006) | pneumonia / influenza | USA | 52% | 58-82* | matched cohort | 19058 | 57174 | inpatient death due to pneumonia, adjusted OR: 0.73 (95% CI 0.47-1.13) |
| Gupta [20]  (2007) | sepsis | USA | 54% | 58 | prospective cohort (dialysis patients) | 143 | 898 | sepsis-related hospitalization incidence rate ratio adjusted for co-morbidities and laboratory values: 0.38 (95% CI 0.21-0.67)  sepsis-related hospitalization incidence rate ratio in the propensity-matched subcohort: 0.24 (95% CI 0.11-0.49) |
| Hackam [21]  (2006) | sepsis | Canada | 56% | 74 | population-based retrospective cohort | 34584 | 34584 | Hazard ratio adjusted: fatal sepsis 0.75 (95% CI 0.61-0.93), severe sepsis 0.83 (95% CI 0.7-0.97), sepsis 0.81 (95% CI 0.72-0.90) |
| Hauer-Jensen [22] (2006) | all infections, postop. | USA | 99% | 62 | retrospective cohort (post op: herniorrhaphy) | 1242 | 9540 | infection: unadjusted OR 1,12 (95% CI 0.51-2.48), our calculation based on extracted crude data. |
| Ishida [23]  (2007) | pneumonia | Japan | ND | >65 | retrospective population-based analysis | NA | NA | correlation between statin sales per capita and pneumonia as major cause of death; R=0.341 (p=0.02) |
| Kwong (2009) [24] | pneumonia | Canada | 45% | 74 | retrospective population-based  matched cohort | 1 120319 | 1 120319 | Pneumonia hospitalization OR 0.92 (95% CI 0.89-0.95)  30-day pneumonia mortality OR 0.84 (95% CI 0.77-0.91) |
| Martin  (2007) [42] | sepsis | USA | 60% | 60 | observational retrospective cohort | 16 | 37 | The risk for septic patients to develop severe sepsis was 30% lower in statin users (56% vs 86%, p<0.02).  in-hospital mortality in septic patients: unadj OR 0.63 (0.19-2.10) ,our calculation based on extracted crude data. |
| Mohamed [25] (2009) | all infections, postop. | Canada | 77% | 65 | secondary analysis of prospective cohort (post op: cardiac surgery) | 2657 | 5076 | adjusted OR: any infection 1.08 (95% CI 0.89-1.31), all-cause death 1.24 (95% CI 0.83-1.87), death due to infection 1.11 (95% CI 0.54-2,28) |
| Montaner [26] (2008) | all infections, post stroke | Spain | 52% | 73 | RCT (patients with acute stroke) | 28 | 28 | infections OR: 2.4 (1.06-5.4) |
| Myles [27]  (2009) | pneumonia | UK | 46% | 70-80* | Population-based case-control | 1228 | 23679 | Pneumonia: adjusted OR 0.78 (95% CI 0.65-0.94) current statin users vs never-users |
| Neal (2009) [28] | MOF,  nosocomial infection | USA | 62% | 67 | Prospective observational cohort study  (injured patients aged > 55 years with hemorrhagic shock) | 71 | 224 | Rates of MOF between SU vs NSU were similar (59.2% vs 47,3%, p=0.08)  Rate of nosocomial infection between SU vs NSU were similar (47,1% vs 52,9%, p=0.399) |
| de Saint Martin (2009) [36] | Fever >38 C | France | 50% | 72 | Prospectice observational cohort study | 139 | 782 | No difference between the groups in mortality rate or lengths of hospitalization. Increased risk of ICU-admission for statin users: relative risk 4.69 (95% CI 2.42-9.08) |
| Schlienger [29] (2007) | pneumonia | UK | 54% | 70-79* | Population-based nested case-control | 150** | 1031** | Death from pneumonia, adjusted OR: 0.47 (95% CI 0.25-0.88), |
| Schmidt [47]  (2006) | MODS | Germany | 73% | 65 | retrospective cohort (ICU patients with MODS) | 40 | 80 | 28-day mortality: Cox proportional hazard analysis: hazard ratio 0.53 (95% CI 0.29-0.99, p=0.04)  MODS death: OR 0.44 (95% CI 0.20-0.96), our calculation based on extracted crude data |
| Smeeth [30]  (2008) | pneumonia / influenza | UK | 50% | 60-69* | Population-based retrospective matched cohort | 129288 | 600241 | pneumonia /death 6 months post flu adjusted hazard ratio 0.84 (99% CI 0.39-1.80) |
| Subramaniam [31] (2008) | all infections, postop. | USA | 76% | 65 | prospective observational cohort (post op cardiac surgery) | 1835 | 662 | serious infections post op: OR 0.68 (95% CI 0.31-1.48) |
| Tseng [32]  (2007) | sepsis, post subarach. hemorrh. | UK | 45% | 53 | RCT (patients with subarach.hemorrh). | 40 | 40 | Sepsis postrandomisation: 15% in the placebo group, 27.5% in pravastatin group (p=0.172).  Reduced mortality related to sepsis: 6.25% vs 71.43%, log-rank test (p=0.001) |
| van de Garde [33] (2006) | Pneumonia | UK | 48% | 70-79* | case-control (diabetic patients) | 372 | 19669 | reduction in risk of pneumonia, adjusted OR 0.49 (95% CI 0.35-0.69) |

Abbreviations: postop. - postoperative, MOF – multiple organ failure, MODS - multiple organ dysfunction failure (due to sepsis, pneumonia, cardiac causes or stroke), RCT - randomised controlled trial, retro cohort - retrospective cohort study, NA - not applicable, subarach. hemorrh. - aneurysmal subarachnoid hemorrhage, UTI-urinary tract infection, CI – confidence interval

*median age or age-interval

**the cases and controls in the “death from pneumonia”-analysis
