# Supplementary material for: Statin Treatment and Mortality in Bacterial Infections – A Systematic Review and Meta-Analysis
Source: PLoS One. 2010 May 19;5(5):e10702. doi: 10.1371/journal.pone.0010702 (PMC2873291; doi:10.1371/journal.pone.0010702)
Supplement: Table S3 — Influence of study characteristics on estimated association between statins and mortality. (0.06 MB DOC) [file pone.0010702.s003.doc]

Table 3: Influence of study characteristics on estimated association between statins and mortality

|  | **Univariable analyses** | | **Multivariable analysis** | |
| --- | --- | --- | --- | --- |
| **Study characteristic** | **Beta** | **p-value** | **Beta** | **p-value** |
| Prospective study | 0.33 | <0.05 | 0.46 | <0.01 |
| Case-control design | 0.03 | ns |  |  |
| Adjustment performed | 0.08 | ns | -0.33 | <0.05 |
| Industry-sponsored | 0.48 | <0.05 |  |  |
| Industry-independent | -0.06 | ns | 0.42 | <0.01 |
| Asian studies | 0.41 | ns | 1.44 | <0.01 |
| European study | 0.07 | ns |  |  |
| North American study | -0.11 | ns |  |  |
| Impact factor of journal | 0.03 | 0.09 |  |  |
| Infection-related mortality | 0.17 | ns | -0.36 | <0.05 |
| In-hospital mortality | 0.01 | ns | 0.49 | <0.01 |
| Age | 0.01 | ns |  |  |
| Proportion men | -0.44 | ns |  |  |
| Pneumonia | 0.03 | ns |  |  |
| Sepsis/bacteremia | 0.04 | ns |  |  |
| Standard error | -0.57 | 0.09 | -0.80 | <0.01 |

Beta indicates the change in OR of death (statin users vs non-users) associated with an increase of the predictor (study characteristic) value by 1. For dichotomous predictors, beta indicates the change in OR associated with presence of the predictor. Since the overall OR was <1, a *negative* beta indicates that the study characteristic is associated with an *increased* apparent statin effect (i.e. a lower OR).
